# Using a New Model of Electronic Health Record Training to Reduce Physician Burnout: A Plan for Action

**DOI:** 10.2196/29374

**Published:** 2021-09-20

**Authors:** Vishnu Mohan, Cort Garrison, Jeffrey A Gold

**Affiliations:** 1 Department of Medical Informatics and Clinical Epidemiology Oregon Health & Science University Portland, OR United States; 2 Division of Pulmonary & Critical Care Medicine Department of Medicine Oregon Health & Science University Portland, OR United States

**Keywords:** electronic health records, clinician burnout, EHR training, clinician wellness, after-hours EHR use, EHR, patient data, burnout, simulation, efficiency, optimization, well-being

## Abstract

Physician burnout in the United States has been growing at an alarming rate, and health care organizations are beginning to invest significant resources in combating this phenomenon. Although the causes for burnout are multifactorial, a key issue that affects physicians is that they spend a significant proportion of their time interacting with their electronic health record (EHR) system, primarily because of the need to sift through increasing amounts of patient data, coupled with a significant documentation burden. This has led to physicians spending increasing amounts of time with the EHR outside working hours trying to catch up on paperwork (“pajama time”), which is a factor linked to burnout. In this paper, we propose an innovative model of EHR training using high-fidelity EHR simulations designed to facilitate efficient optimization of EHR use by clinicians and emphasize the importance of both lifelong learning and physician well-being.

## Introduction

Physician burnout is a significant problem in the United States today. One study has suggested that over 50% of physicians have experienced at least one symptom of burnout; alarmingly, the authors also noted that the frequency of burnout increased by 10% in just three years (2011-2014) [1].

The risk of burnout has only intensified because of the additional stress on physicians caused by the COVID-19 pandemic [2], further underscoring the urgent need to find a way to mitigate this professional crisis.

## Is There a Relationship Between Electronic Health Record Use and Burnout?

While the etiology is multifactorial, the electronic health record (EHR) has been strongly implicated in physician burnout [3], particularly because physicians spend a substantial proportion of their workday using the EHR. For example, primary care physicians spend more than half of their workday (nearly 6 hours) interacting with the EHR, both during and after regular patient care hours [4].

Commercial EHRs tend to be large, complex, clunky software that are often not praised for their ease of use. Needless to say, physician satisfaction with their EHR is generally low [5]. As many as 70% of EHR users have reported health information technology–related stress and a substantial proportion of physicians are unable to complete many of their EHR-related tasks when at work. Therefore, they end up spending an excessive amount of time [6] catching up on EHR “paperwork” (the irony of using this word in the context of the EHR is not lost on us) at home. The implications for this are significant: physicians who reported moderately high or excessive time spent on EHRs at home had almost twice the risk of burnout [7].

## Why Do Physicians Spend So Much Time Catching Up on EHR Work After Hours?

The emphasis on clinical workflow efficiency (a phenomenon that has seen a sharp increase in attention after the advent of the EHR) coupled with the increasing complexity of the medical record has led to an exponential increase in the amount of patient data recorded in the EHR. Not only do primary care physicians spend half their working day at the computer, about half their time in the EHR is spent engaging in clerical and administrative tasks (eg, documentation, order entry, billing, and coding) and about a quarter of the remainder of their EHR time is spent managing their inbox [8]. The clerical burden associated with EHR use, a consequence of compliance and regulatory requirements, may play a key role in promoting physician burnout [9,10]. The amount of EHR time may increase with the inclusion of more genomic and consumer data into the patient record. Combine this with a rapid rise in the use of patient portals due to the COVID-19 pandemic and the result is a “perfect storm” of excessive data and cognitive burden [11]. Some of this can be mitigated by reducing administrative requirements using regulation directed from the federal level and optimizing clinical workflows. However, the continual increase in the information needs of physicians highlights the importance of ensuring that physicians are effectively trained in how to use the EHR to effectively and efficiently perform these tasks, thus minimizing pain points [12,13].

## What Is the Root of the Problem?

One primary issue is that current models of EHR training are limited. As EHR use has become ubiquitous in health care, organizations have typically focused on providing initial training on EHR use to clinicians. These initial training offerings typically focus on basic EHR use but do not provide opportunities to gain workflow proficiency. One study has suggested that 43% of clinician users considered initial EHR training to be “less than adequate” and almost 95% felt that it could be improved [14].

Once they have completed basic EHR training, physicians then learn EHR skills on the job and typically improve their EHR use by the process of trial and error while interacting with the system interface, by gleaning nuggets of best practices from their peers, or by gaining additional insight when there is a significant system update that typically necessitates rolling out a new wave of EHR training. This is not only inefficient, but also offers no certainty that what is gleaned in this on-the-job fashion is truly the best way to use the system.

Additionally, current EHR training models are typically not tailored to a physician’s unique workflow and information needs—in essence, the type of information, the way information is retrieved, and the specifics of documentation are different for, for example, an ambulatory obstetrician, a medical intensivist, and a trauma surgeon. The current model of EHR training, relying on a one-size-fits-all approach, is unable to accommodate for these variations in clinical workflows between specialties and locations.

## What Interventions Have Been Proposed?

Areas for potential interventions include the following: (1) improving EHR-related training, (2) remodeling clinical workflows, and (3) redesigning the EHR to better reflect optimal workflows. In this paper, we present a viable model for transforming EHR education.

Some organizations have attempted to mitigate gaps in EHR training by organizing additional sessions to optimize clinician use of the EHR, either through refresher courses or retreats [15]. One organization has combined this with a structured, rapid assessment of workflow patterns and designed training that is informed by clinician feedback [16]. Others have used one-on-one or group proficiency training to improve self-reported EHR efficiency [10,17], while another organization described an individualized learning plan for physicians to improve their use of the EHR [18].

However, these solutions are, in essence, rescue therapies designed to “undo” the damage of poor initial training. They are also time intensive and for the most part focus on one specific domain, usually centered around improving efficiency in documentation or optimizing charge capture, and do not encompass the full spectrum of EHR activities encountered in a physician’s specialty and workflow.

## What Is Our Model of EHR Training?

### Overview

Over the past few years, we have conducted substantial research in the area of optimizing EHR use using simulations. We pioneered the use of high-fidelity simulation cases that replicate clinical cognitive loads and maintain EHR interface and documentation customizations created by the provider in their clinical environment. This allows learners to use the EHR just as they would when they deliver clinical care [19]. We then developed an intelligent simulation model to facilitate EHR training, which involved the use of an environment that replicated real-world EHR use [20] We also studied clinician interaction with the EHR by using eye tracking systems to assess EHR use during patient simulations [21].

Informed by our research, we have been able to show the utility of EHR-based simulation to improve efficiency and documentation in the patient record in a sustained manner [22] and identify and correct information gathering issues experienced by clinical end users [23]. Another key success factor is the importance of organizational investment in EHR training [24], particularly those that emphasize standards and personalization [25].

**Figure 1:**
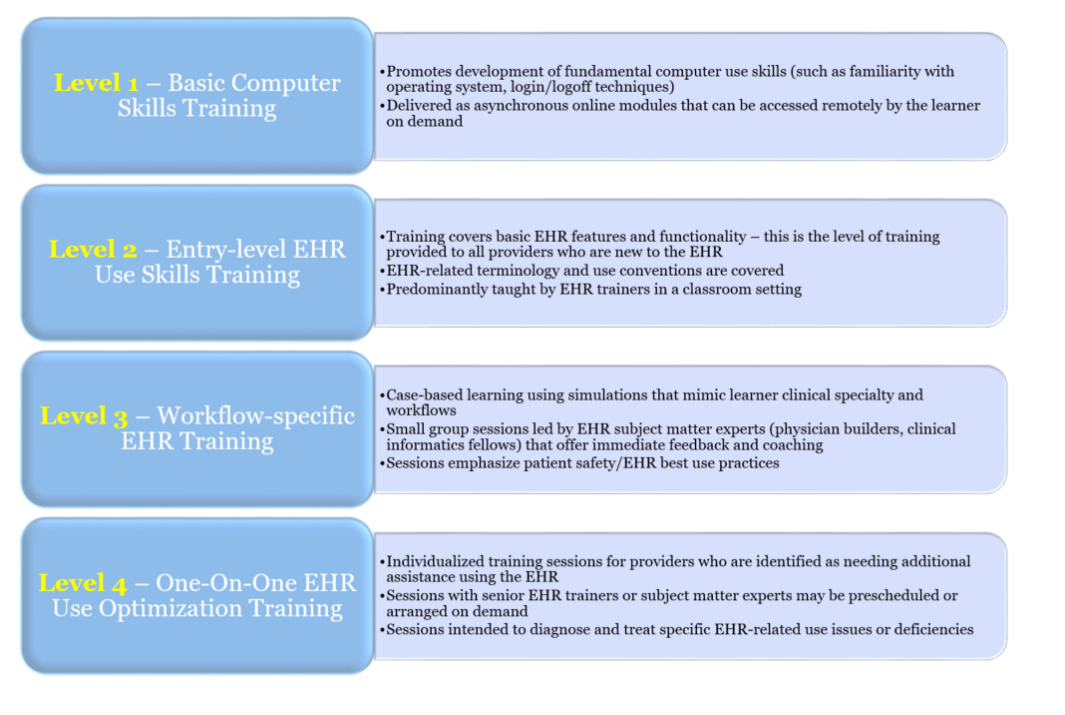
Levels of electronic health record training. EHR: electronic health record.

The results of our research coupled with those of others have allowed us to articulate a model of EHR training that allows efficient optimization of EHR use by clinicians while emphasizing the importance of both lifelong learning and physician well-being (Figure 1).

**Figure 2:**
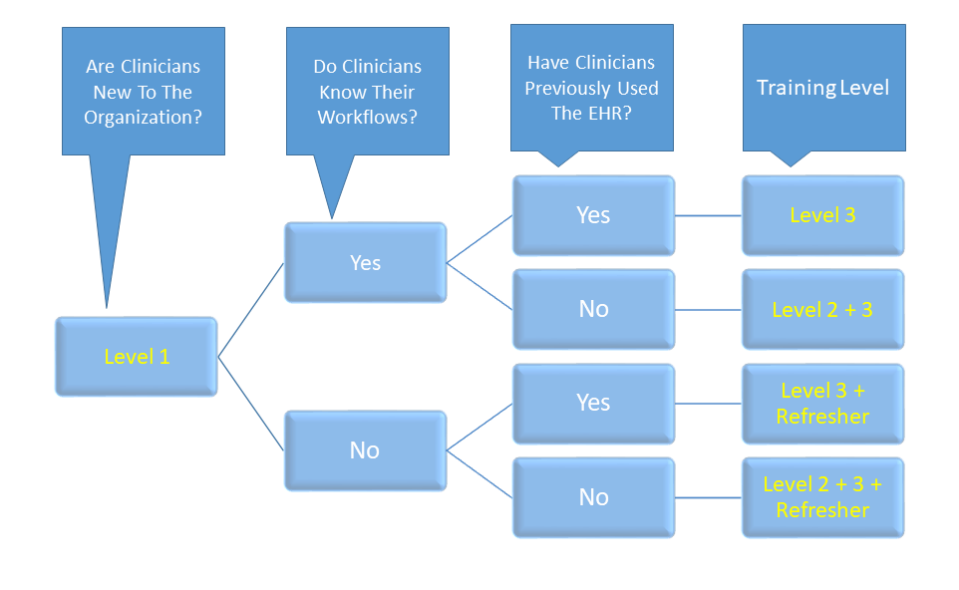
Algorithm for determining level of electronic health record training for physicians. EHR: electronic health record.

The model reorganizes EHR training into four levels, each capable of inducing a progressively higher degree of proficiency with respect to EHR use, and uses a stratified approach to the training process that compensates for prior EHR-related experience and proficiency (Figure 2). We believe that this model represents a paradigm shift in the EHR training universe, one that is more adaptive and responsive to clinician needs. This new model is currently being implemented at our institution.

### Level 1: Basic Computer Training

A surprising number of clinical end users struggle with the EHR because they lack basic computer skills—for example, a provider who exclusively uses Apple products as a consumer may have difficulty navigating the EHR because they are unfamiliar with the Windows operating system. These users may need to participate in learning that builds basic skills. This level of training can easily be delivered asynchronously, using online training modules that learners can complete on their own. Software-based solutions can also be used to correct some basic computer-related deficiencies (eg, improving the speed and accuracy of typing skills).

### Level 2: General EHR Training

Second-level EHR training uses the typical one-to-many instructional model as commonly seen in EHR initial training sessions offered by most health care organizations. These are typically delivered in the classroom by EHR trainers and focus on explanations of basic features and functionality, as well as a demonstration of the fundamentals of EHR navigation, documentation, and order entry. Basic EHR training is an opportunity to emphasize standardized approaches to EHR use; training may also include highlighting high yield screens that clinicians can navigate to in order to optimize their EHR use [26]. Level 2 training is an appropriate entry point for all new users to an EHR system; however, the training should also allow a “test out” option for physicians who may be new to the organization but not to the EHR to avoid repetition of fundamentals.

### Level 3: Workflow-Specific Training Using the EHR

Level 3 training integrates specialty-specific workflows and best practices related to clinical domains and patient safety. This is best achieved by using high-fidelity EHR simulations, where the clinical complexity of the environment can be duplicated in a replicable manner without endangering patient safety, as might occur if using real patient records in a production environment. Small groups of physicians complete simulation-based training sessions led by clinical informaticians, using specialty- and workflow-appropriate patient charts that have been imported into the simulation EHR environment prior to the activity. This model allows for instant debriefing as well as formative and summative feedback and coaching, and promotes retention of concepts learned during the session [22]. Coupling clinically relevant content to workflows familiar to the learner during EHR training is critical to successfully implementing this stage [27].

### Level 4: One-on-one Training and Retraining

This level is characterized by tailored one-on-one EHR training and is typically reserved for providers who are identified as needing additional assistance. Level 4 sessions can be provided on demand or be scheduled to minimally disrupt the provision of clinical care by the learner. We use a simulated clinical case relevant to the provider’s clinical specialty to impart this level of EHR training, coupled with observation and eye tracking and keylogging software to differentiate information retrieval from cognitive issues that the provider may be encountering [23]. The session typically involves running through EHR use activities such as reviewing charts prior to rounding, documenting an encounter, and completing orders. One-on-one observations by the trainer (usually a clinical informatician or expert EHR user familiar with the clinical context) coupled with EHR use data recorded by the eye tracking and/or keylogging software allows the simulation team to effectively analyze EHR use, identify specific deficiencies, and prescribe a bespoke training solution to “diagnose and treat” EHR use issues.

## Final Thoughts

The addition of an instructional designer to the training team greatly improves the quality of learning materials, particularly those that are offered asynchronously. We are obtaining continuing medical education and Maintenance of Certification credits for our EHR training, which promote compliance.

Importation of simulation cases into the training environment can be challenging, and the value of a team member who is trained in data importation into the EHR is critical to the success of any simulation-based training program.

COVID-19 has led to the virtualization of most nonclinical activities conducted by health care organizations, including EHR training. We believe that some forms of simulation-based training can only be provided in the face-to-face setting.

Finally, securing organizational commitment and allocation of adequate resources are often the most challenging elements in developing and deploying a comprehensive EHR training plan, but we believe these are also the most critical factors to ensure success.

## Abbreviations

EHR: electronic health record
